# TRANSITIONING HOME AFTER STROKE: IMPROVING PHYSICAL HEALTH AND PATIENT ACTIVATION

**DOI:** 10.1093/geroni/igz038.2924

**Published:** 2019-11-08

**Authors:** Anne K Hughes, Amanda Woodward, Paul Freddolino, Michele Fritz, Constantinos Coursaris, Sarah J Swierenga, Mat Reeves

**Affiliations:** Michigan State University, East Lansing, Michigan, United States

## Abstract

While the majority of stroke patients will return home after being hospitalized, this transition is physically and emotionally challenging. We developed a social work based case management program to address these challenges. The Michigan Stroke Transitions Trial (MISTT), a pragmatic 3-arm clinical trial tested the effects of the case management program on its own and combined with technology against usual care in patients recovering from stroke. Patients from three Michigan hospitals were randomized to one of three groups upon discharge to home. The two treatment groups received services from a social work case manager via home visit and telephone. One treatment group also was given training and access to a curated stroke website developed for MISTT. The intervention lasted up to 90 days and data was collected via telephone at 7 and 90 days. Quality of life and patient activation were the primary outcomes, measured by the PROMIS Global 10, and the Patient Activation Measure (PAM), respectively. We compared treatment efficacy by comparing the change in outcomes between the three groups (N=265) using a difference-in-differences (D-in-D) analysis. The mean change in PROMIS scores for the social work + technology group was significantly higher than both the social work only group (difference= +2.4; 95%CI=0.46, 4.34; p=0.02) and usual care (difference= +3.4; 95%CI=1.41, 5.33; p<0.001). The mean change in PAM scores for the social work + technology group was significantly higher than the social work only group (+6.7; 95%CI=1.26, 12.08; p=0.02) and marginally higher than usual care (+5.0; 95%CI=-0.47, 10.52; p=0.07).

